# Polysaccharides from Wolfberry Prevents Corticosterone-Induced Inhibition of Sexual Behavior and Increases Neurogenesis

**DOI:** 10.1371/journal.pone.0033374

**Published:** 2012-04-16

**Authors:** Benson Wui-Man Lau, Jada Chia-Di Lee, Yue Li, Sophia Man-Yuk Fung, Yan-Hua Sang, Jiangang Shen, Raymond Chuen-Chung Chang, Kwok-Fai So

**Affiliations:** 1 Department of Anatomy, Li Ka Shing Faculty of Medicine, The University of Hong Kong, Pokfulam, Hong Kong SAR, People's Republic of China; 2 The State Key Laboratory of Brain and Cognitive Sciences, The University of Hong Kong, Pokfulam, Hong Kong SAR, People's Republic of China; 3 Research Centre of Heart, Brain, Hormone and Healthy Aging, Faculty of Medicine, The University of Hong Kong, Pokfulam, Hong Kong SAR, People's Republic of China; 4 School of Chinese Medicine, The University of Hong Kong, Hong Kong SAR, People's Republic of China; 5 Laboratory of Neurodegenerative Diseases, Department of Anatomy, LKS Faculty of Medicine, The University of Hong Kong, Pokfulam, Hong Kong SAR, People's Republic of China; 6 Joint Laboratory for Brain Function and Health (BFAH), Jinan University and The University of Hong Kong, Guangzhou, People's Republic of China; University of South Florida, United States of America

## Abstract

*Lycium barbarum*, commonly known as wolfberry, has been used as a traditional Chinese medicine for the treatment of infertility and sexual dysfunction. However, there is still a scarcity of experimental evidence to support the pro-sexual effect of wolfberry. The aim of this study is to determine the effect of *Lycium barbarum* polysaccharides (LBP) on male sexual behavior of rats. Here we report that oral feeding of LBP for 21 days significantly improved the male copulatory performance including increase of copulatory efficiency, increase of ejaculation frequency and shortening of ejaculation latency. Furthermore, sexual inhibition caused by chronic corticosterone was prevented by LBP. Simultaneously, corticosterone suppressed neurogenesis in subventricular zone and hippocampus in adult rats, which could be reversed by LBP. The neurogenic effect of LBP was also shown *in vitro*. Significant correlation was found between neurogenesis and sexual performance, suggesting that the newborn neurons are associated with reproductive successfulness. Blocking neurogenesis in male rats abolished the pro-sexual effect of LBP. Taken together, these results demonstrate the pro-sexual effect of LBP on normal and sexually-inhibited rats, and LBP may modulate sexual behavior by regulating neurogenesis.

## Introduction

*Lycium barbarum* (commonly known as wolfberry) has been used as an oriental herb in Asian countries for a long history [1]. Being an anti-aging herb, wolfberry has been used for maintaining eye health, nourishing the liver and kidney [2]. Increasing lines of evidence from both clinical and pre-clinical studies support the therapeutic and health-promoting effects of wolfberry. A randomized, placebo-controlled clinical study reveals the consumption of a juice prepared from wolfberry promoted health like improving quality of sleep, mental acuity and decreasing level of fatigue and stress [3]. On the other hand, different pre-clinical studies aim at determining the precise biological activities of wolfberry and the active components exerting the effect. *Lycium barbarum* polysaccharides (LBP), as a major constituent of wolfberry [4], have been shown to exert a wide range of biological effects, including neuroprotection against neurotoxic insults [5], [6], having anti-aging properties in an aging animal model [7], prevention of glaucoma induced by elevated intraocular pressure [4], [8] and immune modulation [9].

Apart from the aforementioned effects, wolfberry was described to exhibit pro-sexual effect by the Chinese herbalist Li Shizhen, and thus wolfberry was included in sexual-enhancing Chinese herbal remedies [10]. Daily consumption of wolfberry juice improves the well-being feeling towards sexuality, including increase in sexual activity and ability [3]. Several studies which focused on the reproductive system showed that LBP was beneficial to male reproduction in several aspects: first, the sexual performance of hemicastrated rats could be improved by LBP treatment [11]; second, the quality, quantity and motility of sperms were increased [12] after LBP treatment;,third, damage of testis and seminiferous epithelium by different insults, such as hyperthermia, H_2_O_2_ and irradiation, were prevented by LBP [10], [11]; and finally, decreases in testosterone of hemicastrated or irradiated rats were reversed by LBP [11], [12]. These findings provided evidence to support the notion that LBP exerts therapeutic effects on male sexual performance and fertility, although the mechanisms underlying the effects remain largely unclear.

**Figure 1:**
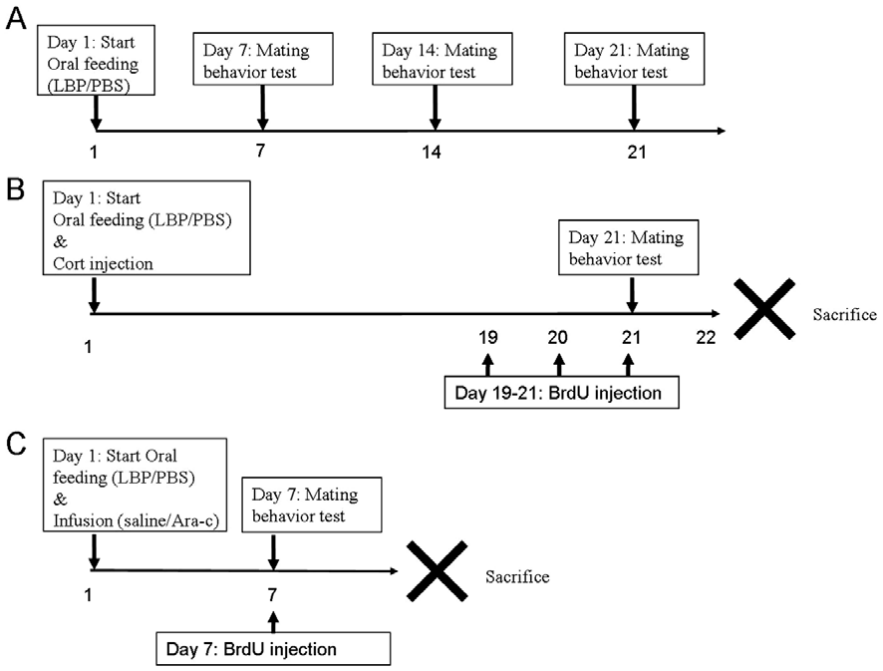
Treatment schedule of experiment 1 (A) and experiment 2 (B). (A): Animals were treated with 0, 1 or 10 mg/kg of LBP daily and sexual behavior tests were conducted at 7-day intervals. (B): The three groups of animals received vehicle (PBS), corticosterone (40 mg/kg) or both corticosterone and LBP treatment for 21 days. Sexual behavior test was conducted at day 21 and was followed by sacrifice of animals at day 22. (C): After intracerebroventricular infusion of either saline (with PBS treatment) or Ara-c (with LBP treatment) for seven days, the animals were subjected to sexual behavior test.

Successful sexual behavior involves complex interplay among different systems including the reproductive system and the nervous system [13]. While previous studies about the effect of LBP on sexual behavior focused on the reproductive system, there is scarce investigation of its influence on the nervous system. The olfactory system and hippocampus in the brain are important regions for perceiving sociosexual stimulus, processing social memory and induce social recognition [13], [14]. These two regions are characterized by their continuous production of adult-born neurons (neurogenesis) during the life. Increasing lines of evidence suggests that neurogenesis may be involved in sexual behavior [15], [16]. For instance, increase in neurogenesis in the subventricular zone (SVZ, a region produces new neurons which migrate to the olfactory bulb) or hippocampus could be found when a female experimental animal was exposed to a male counterpart or sociosexual stimuli, which could be observed in different species like prairie voles [17], mice [13] and ewes [14]. If neurogenesis in the hippocampus and SVZ are blocked, the normal sexual behavior will be disrupted, including the mate-preference in female mice [13] and male mating behavior of rats [18]. Furthermore, the newborn neurons were found to incorporate into the mating-related brain circuit in hamsters [19]. These studies suggest that neurogenesis may have a reciprocal relationship with sexual behavior and the newborn neurons may have an important role in the regulation of sexual behavior.

Given the traditional reputation of wolfberry in promoting sexual performance and the importance of neurogenesis in sexual behavior, we ask 1. whether treatment of LBP could improve male sexual behavior; 2. whether LBP could reverse sexual inhibition; 3. whether LBP affects neurogenesis in *in vivo* and *in vitro* situations and 4. if there is an association between LBP treatment, sexual behavior and neurogenesis. The results will not only provide evidence to support the pro-sexual functions of LBP, but also will provide insight on the biological mechanisms underlying LBP treatment.

**Figure 2:**
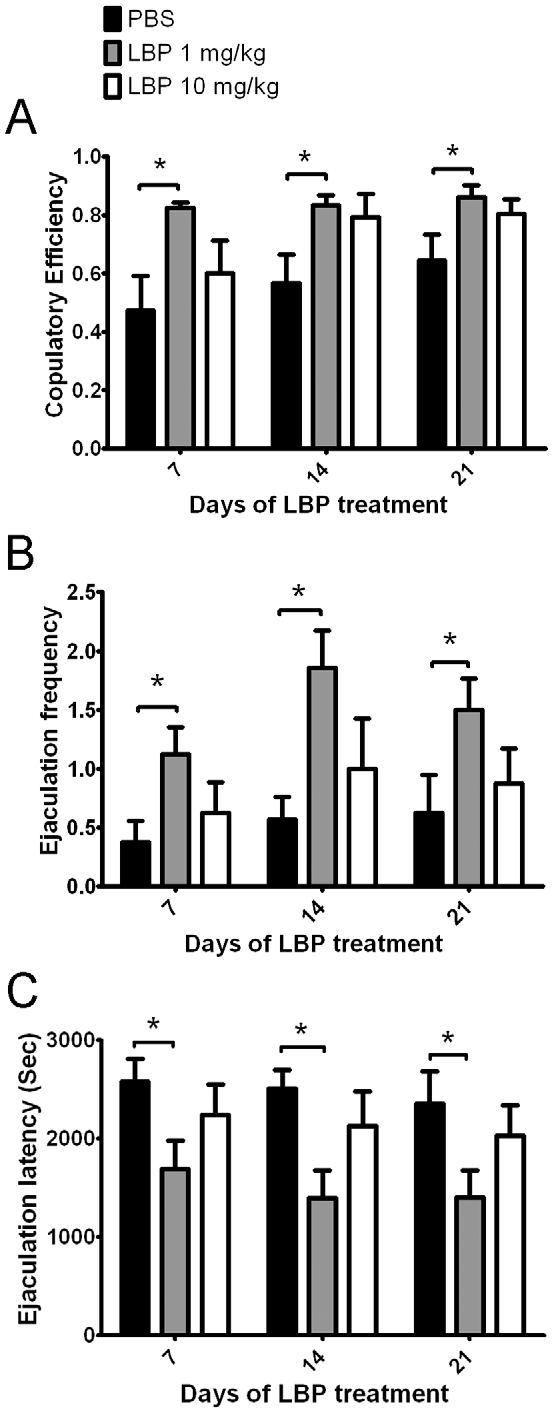
Treatment of LBP at 1 mg/kg improved male sexual behavior in (A) Copulatory Efficiency, (B) Ejaculation Frequency and (C) Ejaculation Latency. No difference was found between vehicle (PBS) group and LBP-treated group (10 mg/kg). Data are presented as mean+SEM. *: p<0.05, ANOVA with LSD post-hoc test.

## Materials and Methods

### Animals and Treatment

The experimental protocol was approved by the animal ethics committee of the University of Hong Kong (Committee on the Use of Live Animals in Teaching and Research; protocol #1961–09). Young adult male Sprague-Dawley rats (280±10 g) were used for the experiment. Animals were kept under a 12/12 light dark cycle at 22°C. Sexually naïve animals were used since disruption of sexual behavior is more obvious in sexually inexperienced animals. Animals were randomly assigned to three experiments: (1) Effect of LBP on sexual behavior at different dosages and time-points; (2) subchronic treatment with corticosterone and LBP and (3) effect of blocking neurogenesis in the pro-sexual effect of LBP. LBP was prepared according to the procedures reported previously [4], [8], [20].

**Figure 3:**
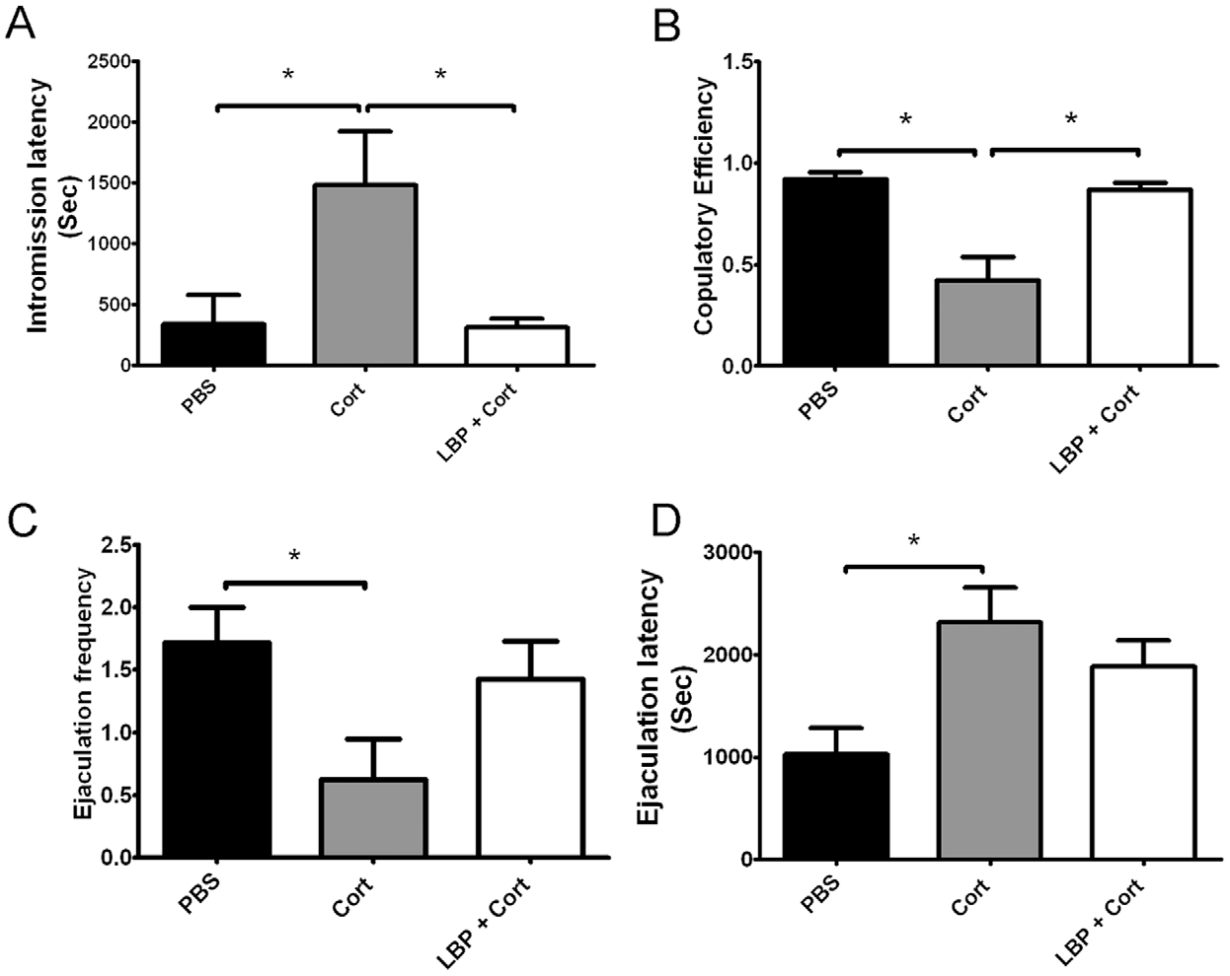
Corticosterone treatment inhibited sexual behavior, which is prevented by LBP treatment. (A): Intromission Latency; (B): Copulatory Efficiency; (C): Ejaculation Frequency; (D): Ejaculation Latency. Data are presented as mean±SEM. *: p<0.05, ANOVA with LSD post-hoc test.

### Treatment Schedule

#### Experiment 1

Animals were divided into three groups. The control group received daily oral feeding of vehicle solution (0.01 M PBS). The other two groups of animals received either 1 mg/kg LBP or 10 mg/kg LBP feeding. The treatment continued for 21 days with the sexual behavior test conducted at day 7, 14 and 21 of the treatment period (Figure 1A).

#### Experiment 2

Animals were divided into three groups. The control group received oral feeding of 0.01 M PBS and subcutaneous sesame oil (vehicle of corticosterone treatment) injection for 21 days. The corticosterone treatment group received subcutaneous injection of 40 mg/kg corticosterone (Sigma, St Louis, MO, USA; suspended in 0.8 mL sesame oil) and oral feeding of PBS daily, while the co-treatment group received both oral feeding of 1 mg/kg LBP and injection of corticosterone during the treatment period. Bromodeoxyuridine (BrdU, Sigma, St Louis, MO, USA) was injected intraperitoneally (50 mg/kg) during the last three days of treatment at 12 h intervals (Figure 1B).

**Figure 4:**
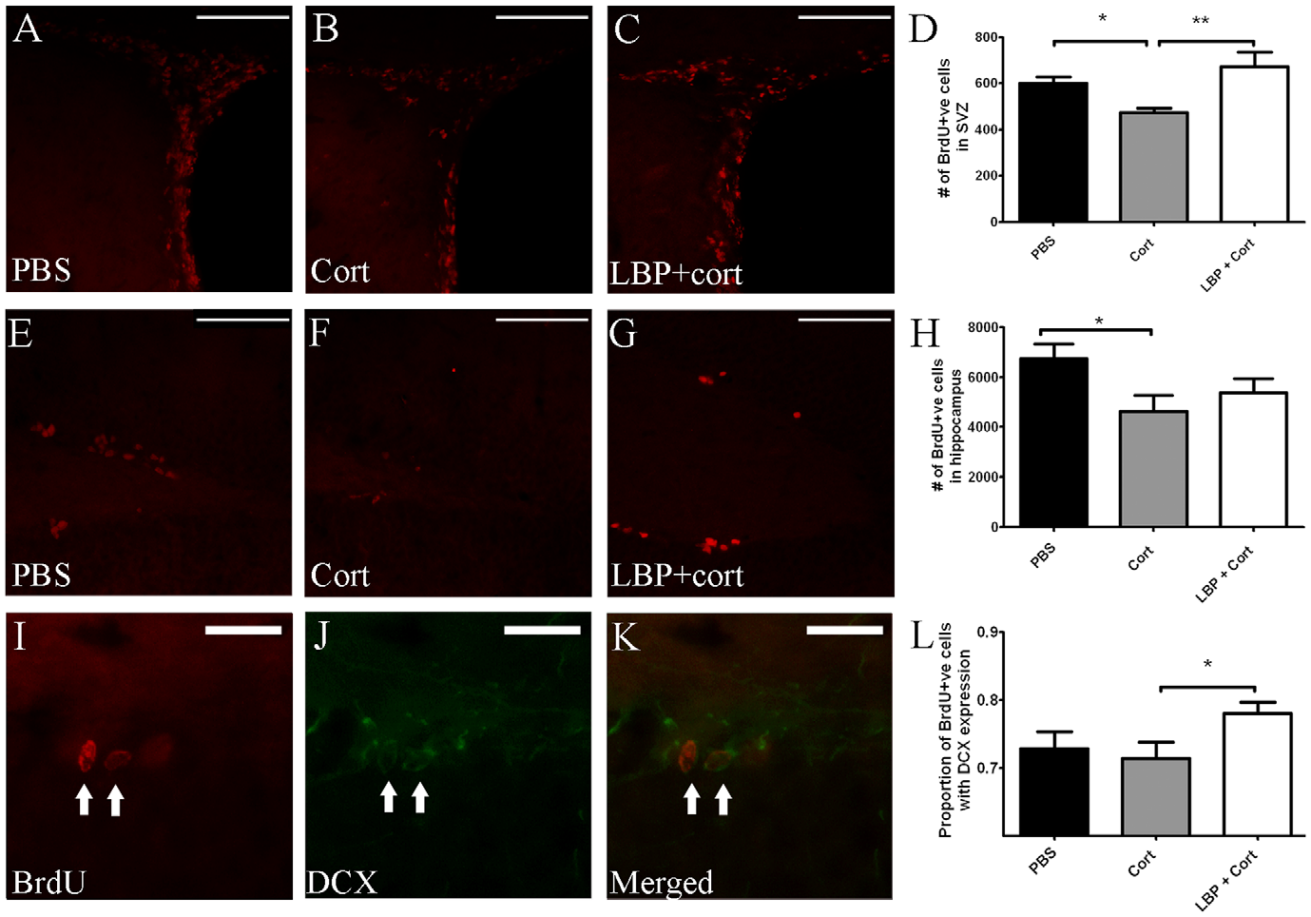
Effect of corticosterone and/or LBP treatment on SVZ and hippocampal neurogenesis. Representative microphotograph of BrdU-positive cells in SVZ of (A) vehicle (B) corticosterone and (C) co-treatment with corticosterone and LBP groups are shown. Corticosterone treatment significantly decreased cell proliferation when compared to vehicle (p<0.05) and co-treatment (p<0.01) groups (D). Representative BrdU-staining of hippocampus are shown in (E [vehicle]), (F [corticosterone]) and (G [co-treatment]). Corticosterone group showed a lower number of BrdU-positive cells than the vehicle group (H, p<0.05). Co-immunostaining of BrdU and DCX experiment (I-K) showed that co-treatment group showed a significantly higher proportion of DCX/BrdU ratio than the corticosterone group (L) Arrows indicates co-labeled cells. Analyzed by ANOVA with LSD post-hoc test. Data are expressed as mean±SEM. Scale bars: A-C & E-G: 50 µm; I-K: 25 µm.

#### Experiment 3

Animals were divided into two groups. The control group received oral feeding of 0.01 M PBS and intracerebroventricular infusion of normal saline. The Ara-c group received oral feeding of 1 mg/kg LBP and infusion of cytosine arabinoside (Ara-c, a cytostatic drug which blocks cell proliferation) for seven days. Procedures of infusion was described previously [21]. In brief, osmotic pumps (Alzet, California, USA) filled with saline/Ara-c were implanted subcutaneously at the dorsal neck region of the rats. Saline/Ara-c was delivered via a cannula, affixed at 1.5 mm lateral and 1.0 mm posterior, to the bregma into the right lateral ventricle. At day 7, BrdU was injected to label proliferative cells and the animals were subjected to sexual behavior testing on the same day (Figure 1C).

**Figure 5:**
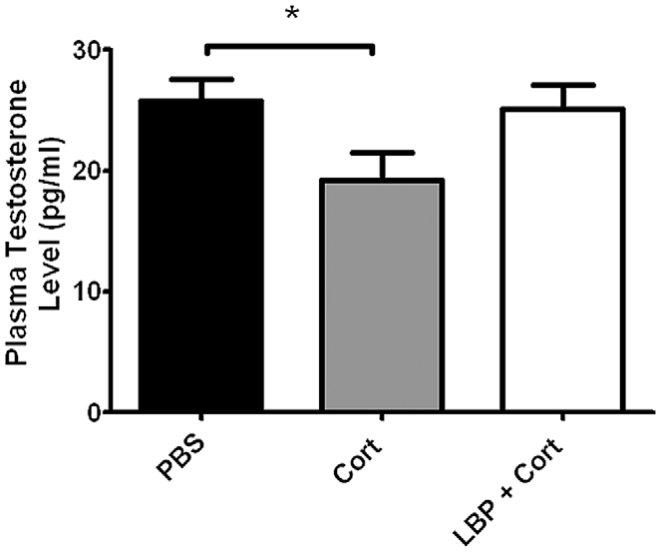
Effect of corticosterone and/or LBP treatment on plasma testosterone level in male rats. Data are represented by mean±SEM. *: p<0.05.

### Sexual Behavior Testing

Female rats (300±20 g, stimulus animals) were induced to become sexually receptive by subcutaneous injection of 50 µg of estradiol benzoate 48 h before testing which was followed by injection of 500 µg of progesterone 4 h before testing. The sexual behavior test was started 2 hours after the onset of dark phase and a dim red light source was used to illuminate the venue at 25–40 lux. Before testing, the subject animals were placed in standard rectangular plastic cages (25 (W) × 28 (D) × 24 (H) cm) for 10 minutes. Then, the sexually receptive females were introduced into the cage and the mating process was video-recorded for 45 minutes for later analysis. The analysis of the sexual performance was done by an experimenter blinded to the treatment conditions. The components observed were: i. mount latency (ML, in seconds): the time between introduction of the female rat and the first mount; ii. intromission latency (IL, in seconds): the time between introduction of the female rat and the first intromission; iii. Mount frequency (MF): number of mount displayed before ejaculation; iv. Intromission frequency (IF): number of intromission displayed before ejaculation; v. Copulatory efficiency (CE): Total number of intromission divided by the sum of intromission and mount frequency; vi. Ejaculation frequency (EF): number of ejaculation during the test session and vii. Ejaculation latency (EL): the time between the first intromission and ejaculation.

**Table 1:** Correlational analysis of BrdU +ve cells in SVZ and hippocampus and sexual behavior components.

	SVZ	Hippocampus
Mount latency	r = −0.583; p<0.01	
Intromission latency	r = −0.57; p<0.01	
Intromission frequency		r = −0.687; p<0.01
Copulatory efficiency	r = 0.5; p = 0.021	
Ejaculation frequency	r = 0.473; p = 0.035	
Ejaculation latency		r = −0.455; p = 0.033

### Tissue Processing and Immunohistochemistry

Animals were deeply anesthetized with an overdose of sodium pentobarbital (100 mg/kg, i.p. injection), and transcardially perfused with normal saline containing 4% paraformaldehyde. After perfusion, the brains were dissected and post-fixed in 4% paraformaldehyde overnight. Cryosections with thickness of 40 µm were prepared in 1-in-12 series using a freezing microtome.

Immunohistochemistry was performed as previous described [22], [23]. After affix on gelatin-coated slides, brain sections were subjected to antigen retrieval (in 0.01 M citrate buffer, pH 6.0 at 85°C) for 25 minutes, followed by incubation in 2 N hydrochloric acid at 37°C for 25 minutes. Then, the sections were incubated with 0.1 M borax buffer (pH 8.5) for 15 minutes and subsequently 10% goat serum for block non-specific antigen binding. Primary antibodies used were mouse anti-BrdU (1∶1000, Roche, Indianapolis, IN, USA) and rabbit anti-doublecortin (DCX, an immature neuronal marker, 1∶300, Cell signaling technology, Beverly, MA, USA). After incubation with primary antibody at room-temperature overnight, sections were then incubated in diluted secondary antibodies (goat anti-mouse Alexa Fluor 568 and goat anti-rabbit Alexa Fluor 488, dilution 1∶200, Molecular Probe, Eugene, OR, USA) at room temperature for 2 h. Sections were examined under a fluorescent microscope (Olympus Bx-52, Tokyo, Japan).

Quantification of BrdU-positive cells in the hippocampus (sections from 2200 µm to 4800 µm posterior to bregma) and subventricular zone (sections from 1800 µm anterior to bregma to 300 µm posterior to bregma) was performed using unbiased stereology. Semi-automated StereoInvestigator (MicroBrightField, Williston, VT, USA) was utilized. For the SVZ, results were expressed as mean of BrdU-positive cells per section while estimated total number of BrdU-positive cells in the whole structure was used for the hippocampus.

**Figure 6:**
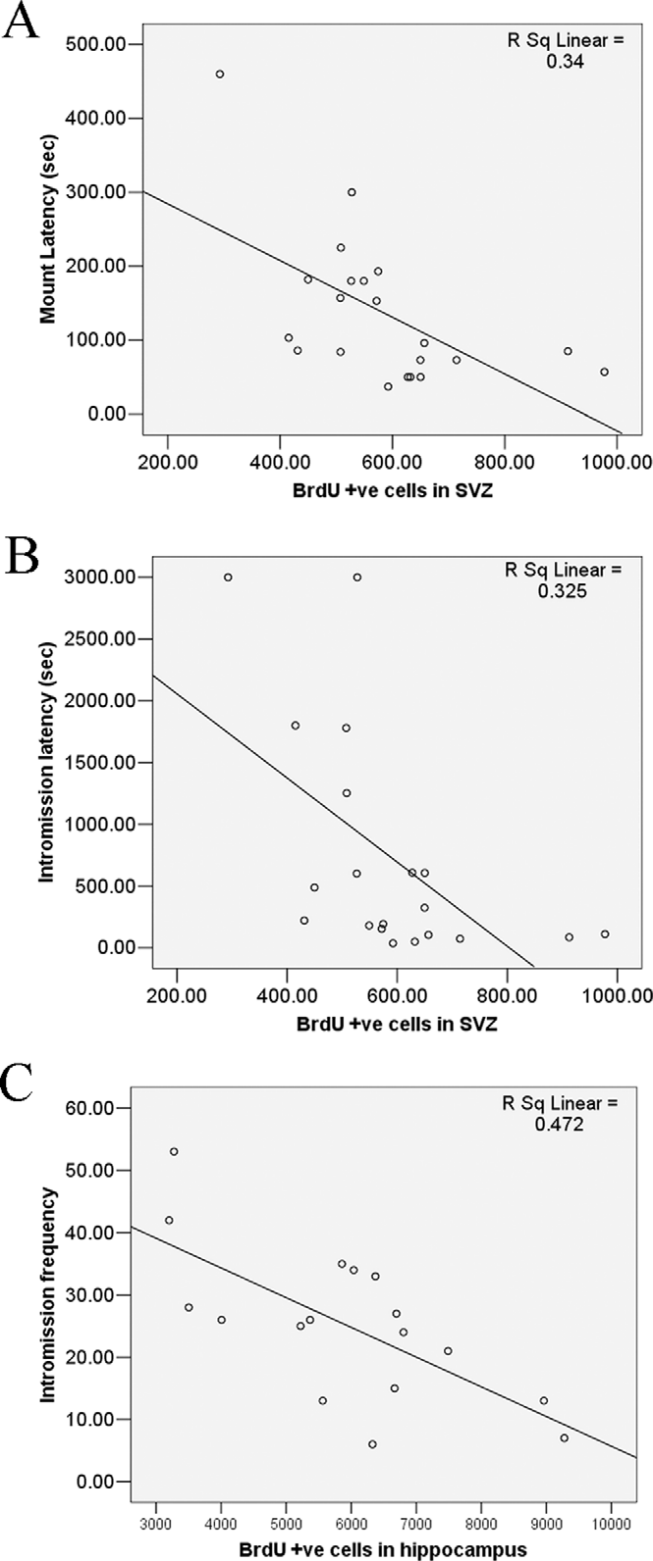
Linear regression of ML and BrdU-positive cells in SVZ (**A**)**, IL and BrdU-positive cells in SVZ** (**B**) **and IF and BrdU-positive cells in hippocampus** (**C**) **in rats treated with PBS, corticosterone or both corticosterone and LBP for 21 days.**

### Enzyme-link Immunosorbant Assay of Testosterone in Plasma

Before transcardial perfusion, truncal blood of rats was collected and heparinized. Plasma was isolated by centrifugation at 3000 g for 15 minutes. The samples were stored at −80°C until the assay was performed. Quantification of plasma testosterone level was carried out using Testosterone EIA kit (Cayman, MI, USA) according to the manufacturer’s protocol.

### In Vitro Neurogenesis Assay

C17.2 neural stem cells were derived from the cerebellum of neonatal mouse and immortalized by retrovirus-mediated v-myc gene transfection [24], [25]. They were routinely cultured to 70–80% confluency in proliferation medium consisting of Dulbecco’s Modified Eagle medium (DMEM, Invitrogen) supplemented with 10% fetal bovine serum (Invitrogen), and L-Glutamine (2 mM, Invitrogen) in standard humidified incubators containing 5% CO_2_ at 37°C.

**Figure 7:**
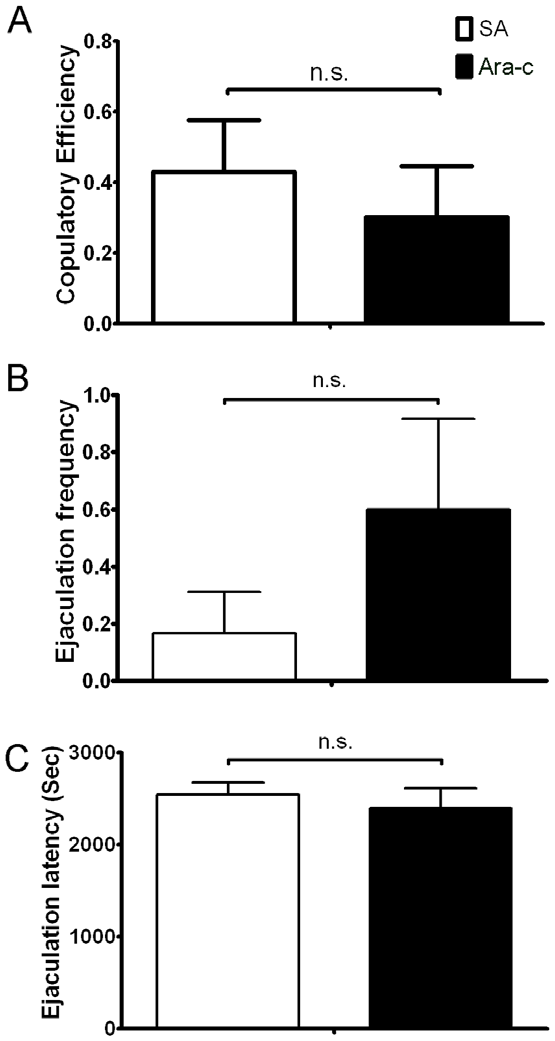
Blocking neurogenesis abolished the pro-sexual effect of LBP treatment. No significant difference between saline infused-animals (SA) and LBP-treated animals with Ara-c infusion (Ara-c) in (A) Copulatory Efficiency, (B) Ejaculation Frequency and (C) Ejaculation Latency was found. Data are presented as mean+SEM. *: p>0.05, Student’s *t*-test.

For BrdU labeling, NSCs were seeded and plated onto coverslips in 24-well plate (104 cells per well) in culture medium consisting of Dulbecco’s Modified Eagle medium (DMEM, Invitrogen) supplemented with 2%fetal bovine serum (Invitrogen), and L-Glutamine (2 mM, Invitrogen) in the presence of LBP (1 µg/mL, 10 µg/mL) and/or 1 µM corticosterone (Cort). After 44 h, BrdU (10 µM, Sigma) was added and the incubation was performed for an additional 4 h. NSCs were fixed for 20 min with 4% paraformaldehyde, washed three times with PBS (pH 7.2), and treated with 2 M HCl for 30 min at 37°C. Cells were washed twice with PBS, blocked and permeabilized in 10% normal goat serum plus 0.1% Triton X-100 for 1 h at room temperature. After blocking, the cells were washed with PBS and then incubated in primary rat-anti BrdU (1∶200, AbDSerotec) at 4°C overnight. After rinsing with PBS, the coverslips were incubated with appropriate species-specific Alexa Fluor 488-conjugated IgG (1∶200, Invitrogen) antibody in the dark at room temperature for 1 h. The coverslips were then incubated in nuclei counterstained with DAPI (4,-6-Diamidino-2-phenylindole, 1 µg/ml) and mounted in fluorescent mounting medium (Dako). The fluorescence imaging was visualized by using a Carl Zeiss Axio Observer Z1 fluorescent imaging system. The numbers of total and BrdU-positive cells were counted using microscopy in six non-overlapping fields per coverslip. Results were expressed as relative percentage of BrdU-positive cells. The images obtained were analyzed by mean of Image J software (NIH, USA).

### Statistical Analysis

Comparison among the different treatment groups were conducted using One-way ANOVA, with LSD post-hoc test if required. A statistically significant difference was indicated when p≤0.05. Data are expressed as mean ± SEM.

**Figure 8:**
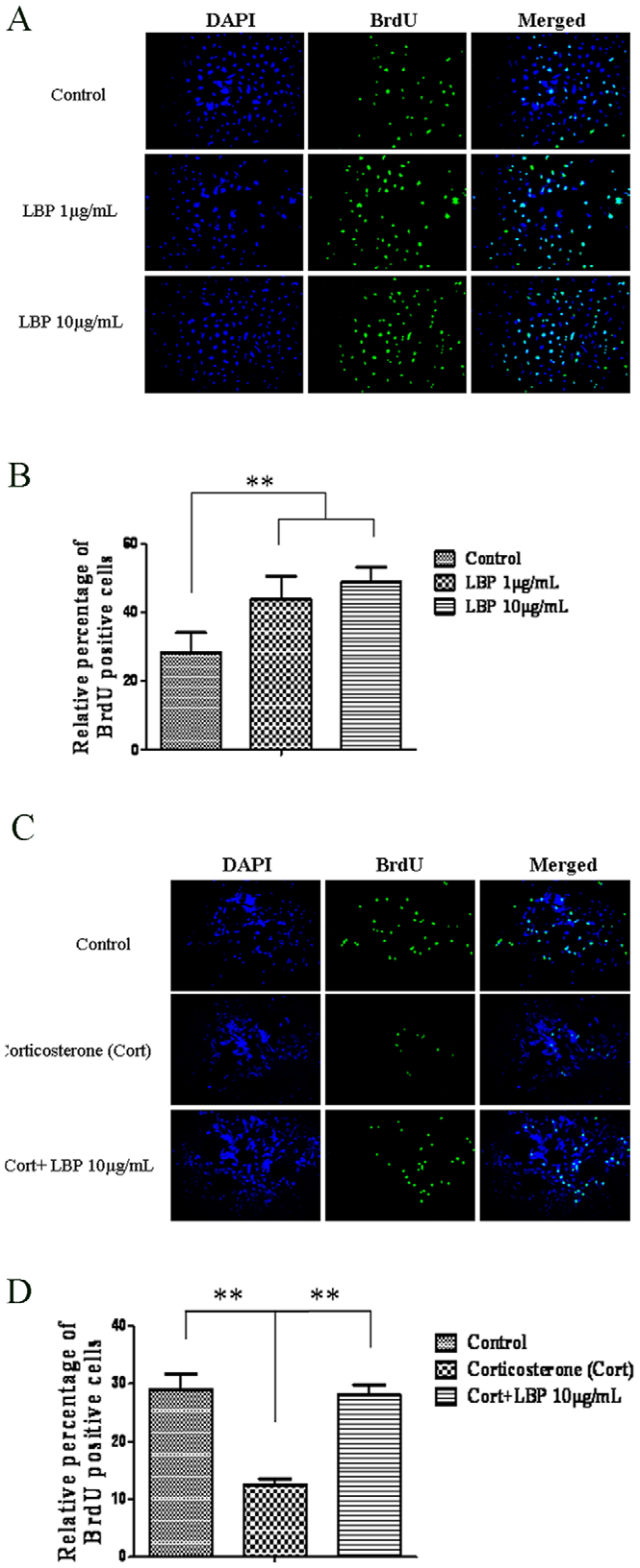
LBP increased cell proliferation in C17.2 neural stem cell line and reversed the suppressed neurogenesis by corticosterone treatment. LBP treatment at 1 µg/ml and 10 µg/ml increased cell proliferation when compared to control group (A & B). While corticosterone (1 µM) suppressed cell proliferation, co-treatment with LBP (10 µg/ml) reversed the suppression. Data are presented as mean±SEM; **: p<0.01.

## Results

### LBP Facilitated Male Sexual Behavior

There were no significant differences in the ML, MF, IL and IF found when comparing the three groups of rats treated with different dosages of LBP (vehicle, 1 mg/kg and 10 mg/kg) at any time-point (data not shown).

However, as shown in figure 2, ANOVA followed by post-hoc test detected significant difference in the CE (Figure 2A), EF (Figure 2B) and EL (Figure 2C) between the vehicle-treated and LBP (1 mg/kg)-treated groups. The LBP (1 mg/kg)-treated animals showed a significantly higher CE (Figure 2A) and EF (Figure 2B) than vehicle-treated animals, while EL (Figure 2C) was significantly decreased by the treatment (p<0.05 for all comparisons). The differences were found in all sexual behavior tests at day 7, 14 and 21. In contrast, no difference was found when comparing the LBP-treated group to vehicle group at dosage of 10 mg/kg.

### LBP Reversed Sexual Behavior Inhibition Induced by Corticosterone

Corticosterone resulted in decreased sexual performance when compared to vehicles-treated control animals in terms of significantly longer IL (Figure 3A), lower CE (Figure 3B), lower EF (Figure 3C) and longer EL (Figure 3D, p<0.05 for the comparisons). Co-treatment with LBP (1 mg/kg) significantly reversed the inhibited sexual behavior, which is indicated by a significantly lower IL (Figure 3A) and higher CE (Figure 3B) than that of the corticosterone group (p<0.05). Although no significant difference was found between the corticosterone group and co-treatment group in EF and EL, the co-treatment group did not show difference from the vehicle-treated group (Figure 3C and 3D).

### LBP Reversed Suppressed Adult Neurogenesis Induced by Corticosterone

In the SVZ, the number of BrdU-positive cells in the corticosterone-treated animals was significantly lower than both vehicle or co-treatment groups (Figure 4 A-D; p<0.05). Corticosterone also decreased the number of BrdU-positive cells in the hippocampus (Figure 2E-H, p<0.05). No difference was found between the co-treatment group and vehicle group. The proportion of DCX-expressing new cells (i.e. ratio of DCX/BrdU) was significantly higher in the co-treatment group when compared to vehicle or corticosterone groups (Figure 4I-L, p<0.05).

### Corticosterone Suppressed Plasma Testosterone Level, which is Prevented by LBP Treatment

Statistical analysis showed a significant decrease in plasma testosterone level of corticosterone-treated animals (p = 0.037 compared to PBS group). No significant difference could be found between the PBS and Cort+LBP groups (Figure 5).

### Adult Neurogenesis in SVZ and Hippocampus were Correlated with Sexual Performance

When the sexual behavior data of the PBS, Cort and LBP+Cort treated groups were plotted against the BrdU+ve cells in the SVZ and hippocampus, significant correlations were found (Table 1). Graphical representations of ML against BrdU positive cells in the SVZ, IL against BrdU positive cells in the SVZ and IF against BrdU positive cells in the hippocampus were shown in figure 6. In general, high number of BrdU positive cells in both regions was associated with a better sexual performance.

### Blocking Neurogenesis Abolished the Pro-sexual Effect of LBP

To determine the direct relationship between neurogenesis and sexual behavior, neurogenesis in animals treated with LBP were blocked with Ara-c infusion. Quantification of BrdU-positive cells in the SVZ and hippocampus of control animals (SA group: saline infusion and PBS feeding) and LBP/Ara-c treated animals (Ara-c group) showed that cell proliferation was significantly inhibited in the SVZ (SA: 618.3±32.7; Ara-c: 74.5+2.34; p<0.01) and hippocampus (SA: 8501.5±551.6; Ara-c: 1561.7+283.8; p<0.01) of Ara-c group. In contrast to experiment 1, blocking neurogenesis in LBP-treated animals did not result in improvement of sexual behavior in copulatory efficiency (Figure 7A), ejaculation frequency (Figure 7B) or ejaculation latency (Figure 7C).

### LBP Increased Neural Stem Cell Line Proliferation and Reversed the Suppressive Effect of Corticosterone in Vitro

In vitro study showed that LBP treatment at 1 µg/ml and 10 µg/ml increased cell proliferation in neural stem cell line C17.2 (Figure 8A & B, p<0.01). Similar to the results depicted in experiment 2, corticosterone treatment suppressed the cell proliferation of C17.2 cell line, while co-administration with LBP at 10 µg/ml could reverse the suppression (Figure 8C & D, p<0.01).

## Discussion

The present study demonstrated the enhancing effect of LBP on male sexual behavior in rats. In both experiments 1 and 2, the CE, EF and EL were improved by LBP and only IL was also improved in experiment 2. The effects of LBP appeared to enhance both motivation (indicated by IL) and copulatory performance (indicated by CE, EF and EL) [26]. Furthermore, it is shown that the improved sexual performance was associated with increased neurogenesis in the two neurogenic zones, suggesting that neurogenesis may be associated with the sexual-enhancing effects of LBP. The causal relationship between neurogenesis and sexual behavior was demonstrated in experiment 3, in which the sexual-enhancing effect of LBP was abolished by blocking neurogenesis.

Wolfberry has been prescribed for treating male infertility for a long history, although there is a scarcity of scientific evidence for this indication [10]. Recently a study revealed that an improved subjective view on sexual ability was found in subjects chronically consumed a juice prepared from wolfberry [3]. This study suggests the potential clinical use of wolfberry to improve male sexual performance.

The findings of the present study showed the sexual-enhancing effect of LBP from a pre-clinical point of view. Our results showed that the beneficial effect was not only revealed in normal, healthy rats, but also in rats with sexual inhibition induced by corticosterone. The ‘rescuing’ effect of LBP on male sexual behavior suggests that LBP may be useful to treat male sexual dysfunction. This notion agrees with previous studies in which castration and irradiation on reproductive tissues suppressed male mating behavior, while LBP improved the mating ability of the affected rats [11], [12]. Apart from behavioral aspect, LBP may exert its beneficial effects on the reproductive system at cellular level. For example, LBP was found to protect spermatogenic cells from apoptosis induced by hyperthermia and the structural integrity of the seminiferous epithelium could be preserved by LBP [10], [11]. In short, the sexual-enhancing effect of LBP may be multi-faceted, ranging from protection of gametes against harmful insults to enhancement of individual sexual behavior. Thus, how does LBP benefit reproduction may warrant further investigations.

The relationship between neurogenesis and sexual behavior shown in this study suggests that neurogenesis may be involved in copulation, and neurogenesis could be one of the mechanisms of how LBP exerts its pro-sexual effect. Neurogenesis could be found at different brain regions while the two most prominent neurogenic regions are the SVZ and hippocampus [27], [28]. Neural progenitor cells proliferate at the SVZ and migrate tangentially along the rostral migratory stream (RMS) to reach the olfactory bulb, which allows continuous addition of new neurons for formation of new circuits throughout the adulthood in rodents [29]. The renewal of interneurons in the olfactory bulb renders the ability of olfactory learning, including differentiating sex-related olfactory signals [30]. As the hippocampus is well recognized for its necessity in memory formation[31], it may also take part in the formation of memory regarding the processing of sexual cues [32].

Previous studies outlined the interrelationship between adult neurogenesis and sexual behavior. For instance, exposure of female prairie voles or ewes to males increased neurogenesis in the SVZ and hippocampus [14], [17]. Another study showed that newborn cells in the olfactory bulbs integrated into the ‘mating’ circuit and could be activated by estrus female pheromones [19]. More recent studies showed that neurogenesis is essential for the display of normal sexual behaviors in both female and male rodents [13], [18]. Under normal situation, female mice prefer to mate with dominant males. When the neurogenesis in the SVZ and hippocampus were blocked by cytostatic cytosine arabinoside, the preference was then abolished [13]. On the other hand, when neurogenesis was suppressed in male rats, the sexual performance was impaired [18]. The decreased neurogenesis was associated with a decrease in neuronal activation in mating-related circuits, which may be the reason underlying the sexual inhibition. The present study further showed that blocking neurogenesis could abolish the sexual-enhancing effect of LBP. These observations indicate that adult neurogenesis warrants the reproductive success of rodents, and disruption of it would cause dysfunction in sexual behavior. In the present data, corticosterone suppressed both neurogenesis and sexual performance simultaneously while LBP could prevent these from occurring. The neurogenesis promoting effect of LBP was further confirmed in the *in vitro* assays, in which the corticosterone-induced proliferation inhibition [33] was reversed by LBP. Apart from cell proliferation, LBP also promotes the neuronal differentiation of neural precursor cells as shown by DCX staining. The neurogenic effect of LBP agrees with previous findings [34]. Interestingly, the sexual performance was found to correlate with neurogenesis. These findings provide further evidence to support the notion that the newborn neurons participate in sexual behavior. Furthermore, neurogenesis in the SVZ and hippocampus are correlated with different measurements in the sexual behavior test, which implies that SVZ neurogenesis may be intimately involved in sexual motivation (indicated by ML/IL) while both the SVZ and hippocampus are involved in copulatory performance. Further studies are required to explore this possibility and this may provide insight on the neural basis of reproductive biology.

Previous studies have shown a dose-dependent response of LBP treatment on neuroprotection [8]and sexual behavior [11]. We choose the current dosages (1 mg/kg and 10 mg/kg) since it was reported that the maximum therapeutic effect could be achieved by these two dosages [6], [8], [11]. Previous reports showed that high dose of LBP inhibited c-jun N-terminal kinase (JNK) pathway [20], 35, which may subsequently inhibit neural stem cell proliferation [36]. This may be the potential mechanism of loss of neurogenesis and sexual-enhancing effect of LBP at high dosage, while this would be confirmed in future studies.

It should be noted that neurogenesis consists of different stages including cell proliferation, differentiation, migration, survival and incorporation of new neurons into the existing neural circuit [15]. The current study investigated the early phases including cell proliferation and differentiation, while later phases were not studied. While newly proliferated cells may act as a source of trophic factors which affect functioning of other neurons, much matured new neurons may directly involve in signal transduction. Further studies of the effect of LBP on the later phases, which may extend the treatment period to over eight weeks, may suggest its long term effect on sexual behavior of the animal from the view of neurogenesis.

LBP may also modulate sexual behavior via increasing the expression of testosterone in sexually inhibited animals. Our results show that chronic corticosterone significantly suppressed the plasma level of testosterone; and thus resulting in inhibited sexual performance. Concomitance to enhancing the sexual performance, LBP also reverse the decrease in testosterone level. Similar result was found by Luo et. al. [11], [12] that LBP increased the testosterone levels in rats subjected to castration or irradiation on testis. Alternatively, as it is recently reported that antioxidant could increase the sexual motivation [37], LBP may enhance sexual behavior through its antioxidant property [38].

To conclude, this study provides evidence for the pro-sexual effect of LBP in both normal animals and animals with suppressed sexual performance. Such findings support the use of wolfberry as a treatment for sexual dysfunction or as an aphrodisiac treatment. LBP could also reverse the decreased neurogenesis induced by corticosterone, which is a possible mechanism underlying its effect. Further exploration on the mechanisms would provide stronger rationale for the use of wolfberry in clinical situations. Furthermore, the effect of wolfberry on female sexual function is not yet explored. Future test of LBP on female rodent models may provide insight on the potential usage of wolfberry on treating female sexual dysfunction, such as sexual desire or arousal disorders.
